# Estimation of horizontal running power using foot-worn inertial measurement units

**DOI:** 10.3389/fbioe.2023.1167816

**Published:** 2023-06-22

**Authors:** Salil Apte, Mathieu Falbriard, Frédéric Meyer, Grégoire P. Millet, Vincent Gremeaux, Kamiar Aminian

**Affiliations:** ^1^ Laboratory of Movement Analysis and Measurement, École Polytechnique Fédérale de Lausanne, Lausanne, Switzerland; ^2^ Digital Signal Processing Group, Department of Informatics, University of Oslo, Oslo, Norway; ^3^ Institute of Sport Sciences, University of Lausanne, Lausanne, Switzerland; ^4^ Sport Medicine Unit, Division of Physical Medicine and Rehabilitation, Swiss Olympic Medical Center, Lausanne University Hospital, Lausanne, Switzerland

**Keywords:** biomechanics, machine learning, wearable sensors, movement analysis, signal processing, quantitative feedback

## Abstract

Feedback of power during running is a promising tool for training and determining pacing strategies. However, current power estimation methods show low validity and are not customized for running on different slopes. To address this issue, we developed three machine-learning models to estimate peak horizontal power for level, uphill, and downhill running using gait spatiotemporal parameters, accelerometer, and gyroscope signals extracted from foot-worn IMUs. The prediction was compared to reference horizontal power obtained during running on a treadmill with an embedded force plate. For each model, we trained an elastic net and a neural network and validated it with a dataset of 34 active adults across a range of speeds and slopes. For the uphill and level running, the concentric phase of the gait cycle was considered, and the neural network model led to the lowest error (median ± interquartile range) of 1.7% ± 12.5% and 3.2% ± 13.4%, respectively. The eccentric phase was considered relevant for downhill running, wherein the elastic net model provided the lowest error of 1.8% ± 14.1%. Results showed a similar performance across a range of different speed/slope running conditions. The findings highlighted the potential of using interpretable biomechanical features in machine learning models for the estimating horizontal power. The simplicity of the models makes them suitable for implementation on embedded systems with limited processing and energy storage capacity. The proposed method meets the requirements for applications needing accurate near real-time feedback and complements existing gait analysis algorithms based on foot-worn IMUs.

## 1 Introduction

Mechanical power generated during running is a measure of the intensity of the run. As an indicator of intensity, power can be used to augment external load monitoring for training programs and to develop pacing strategies for competitions. A reduction in running power for a constant running speed indicates a decrease in aerobic power and thus an improvement in running economy (Taboga et al., 2021). Internal factors, such as fatigue, stress, and hydration, or environmental factors, such as humidity, temperature, and presence of competitors, can influence the perception of internal load and heart rate response (Halson, 2014). Since running power is not directly affected by these factors, it can serve as a useful additional metric for monitoring the training load (Paquette et al., 2020). Unlike heart rate, which is affected by cardiac drift and has a higher response latency (Billat et al., 2020), power provides an immediate measure of running intensity and can thus potentially help optimize pacing strategies. In cycling, the widespread use of mechanical power as a tool for optimizing training adaptation has been facilitated by the availability of reliable power meters (Erp et al., 2019). Since the crankshaft force and speed can be measured directly, mechanical power can be measured with sensors integrated into the bicycle (Passfield et al., 2017). However, such a direct measurement of force and speed during real-world running is challenging.

Mechanical power is defined as the time derivative of mechanical work or the rate at which work is performed. Thus, quantification of mechanical work during running provides a way for estimating the power. Different in-lab approaches have been proposed to measure the total mechanical work produced by the body and derive the power for level running over a range of speeds (Cavagna et al., 1964; Cavagna and Kaneko, 1977; Williams and Cavanagh, 1987; Rabita et al., 2015; van der Kruk et al., 2018). Mechanical work is classified into two types: internal work, i.e., the work carried out in moving the limbs with respect to the center of mass (CoM) of the body and external work, which results from the movement of CoM of the body with respect to the environment (Cavagna and Kaneko, 1977). Limb motion is usually measured with marker-based motion tracking systems, whereas CoM kinetics and ground reaction forces (GRFs) additionally require the use of force plates. When comparing estimated mechanical power at similar speeds, existing approaches based on these instrumentation resulted in different findings, and an universally accepted approach has not been established (Arampatzis et al., 2000; Winter et al., 2016; van der Kruk et al., 2018). However, The inclusion of GRF and running speed in the estimation of work and power improved accuracy and was consistent with expected increase in power due to an increase in running speed (Arampatzis et al., 2000). The incline of the running surface may influence speed and GRF and possibly running power (Wickler et al., 2000). Therefore, the GRF, running speed, and incline of the running surface can be considered together as a reference system for estimation. However, accurate measurement of GRF with force plates is impractical under real-world running conditions.

Wearable inertial measurement units (IMUs) have been used to estimate peak braking GRF (Neugebauer et al., 2014). However, the complete antero-posterior GRF profile is essential for the estimation of mechanical work (and power) involved in push-off and braking phases (Arellano and Kram, 2014). Furthermore, these estimations of GRF have been validated for level running and may not show similar performance under uphill and downhill conditions. Of the commercially available body-worn devices, studies recommend the foot-worn Stryd™ device due to its high repeatability of measurements and its concurrent validity (r ≥ 0.911; SEE ≤7.3%) with respect to the VO_2_ values (Cerezuela-Espejo et al., 2021). One study reports the power estimated by the Stryd device for different treadmill speeds during level running to reflect (mean difference M.D.: −1.04 W kg^-1^; limits of agreement L.O.A: −2.3 to 0.18 W kg^-1^) the reference power measured as a dot product of horizontal (in the direction of running) and vertical forces and velocity, respectively, obtained from a force plate (Taboga et al., 2021). Another study, however, reports an underestimation of power from the Stryd™ device (Imbach et al., 2020). Furthermore, this system has not been validated for running on slopes, which is an important requirement for trail running or long-distance races. Finally, the estimated power output has shown inadequate changes in response to intentional changes in running technique and temporal parameters (Baumgartner et al., 2021), such as step frequency (±10% change), contact time (∼ ±20 ms), and arm swing (presence/absence). Analytical models have focused either on the characterization of the overall race performance (Mulligan et al., 2018) or only on the power requirement while running on flat terrain (Jenny and Jenny, 2020). An approach based on simulated wearable IMUs has shown promise (RMS error range 4.2%–20.1%) but requires data from 15 body segments (Fohrmann et al., 2019). In this study, IMU data were simulated with the virtual acceleration and angular velocity values obtained from a full-body marker-based motion capture system. Neither of these power estimation approaches are suitable for accurate near real-time feedback in the field.

Given the potential of body-worn IMU and global navigation satellite system (GNSS) to estimate running speed (Apte et al., 2020; Falbriard et al., 2021), the relationship between mechanical power and running speed (García-Pinillos et al., 2019) could be used to predict power. However, this relationship is affected by terrain slope and running technique. Terrain slope can be estimated using accelerometer signals (Herren et al., 1999) or a barometer (Moncada-Torres et al., 2014), while the running technique can be characterized by spatiotemporal gait parameters. One such parameter is the vertical stiffness of the spring-mass model used to simulate running, which explains the higher efficiency of running movement that far exceeds analytic muscle efficiency (Cavagna et al., 1964). Although vertical stiffness cannot be measured directly under real running conditions, it can be estimated indirectly using spatiotemporal parameters such as contact time, flight time, and running speed. Previous research has presented an accurate assessment of these parameters (Falbriard et al., 2018; 2020) and their application under real-world conditions (Apte et al., 2022; Prigent et al., 2022) using foot-worn IMUs.

The current study aims to extend this work by estimating horizontal running power during level and graded running at different running speeds. Here, horizontal power is defined only through the components of force and velocity in the running direction and thus directly relates to the ability of the athletes to produce higher propulsion (Jaskólski et al., 1996). This definition is useful for application as a feedback tool for measuring and optimizing running intensity during training and competition and can be tracked longitudinally across multiple training sessions to measure improvements in the capacity of runners (Aubry et al., 2018; Paquette et al., 2020). With a single IMU on each foot, we aimed to achieve similar, if not better, performance (RMS error range 4.2%–20.1%) to that obtained with a simulated whole-body setup IMU (Fohrmann et al., 2019). We considered approaches based on machine learning because of their demonstrated potential for unobtrusive analysis of running that can identify movement-specific risk factors for injury (Franklyn-Miller et al., 2017), accurate estimation of running speed, GRFs, and lower extremity kinematics (Wouda et al., 2018; Gholami et al., 2020; Falbriard et al., 2021), and identification of movement deficiencies (Richter et al., 2019) (Xiang et al., 2022). The various situations considered here include a range of running speeds and inclines, knowledge of which will serve as complementary information to that obtained from IMU signals. In addition, the models proposed here are intended to be computationally inexpensive to allow their use in near real-time power estimation with conventional embedded electronic devices.

## 2 Methods

### 2.1 Materials and measurement protocol

**FIGURE 1:**
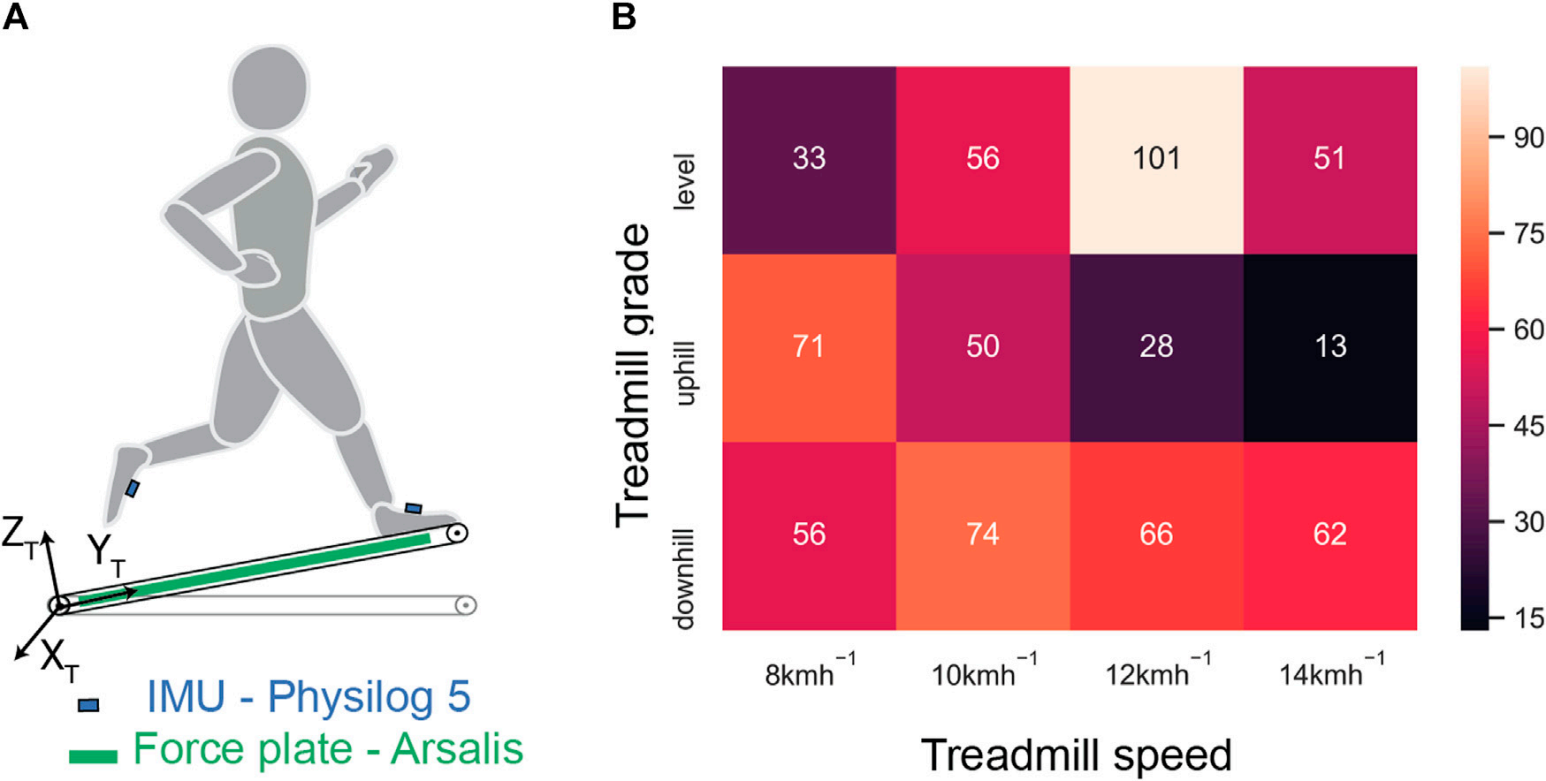
Measurement systems and protocol. **(A)** An IMU was attached to each foot, and force plate data were used as the reference. X_T_-Y_T_-Z_T_ represents the frame of reference attached to the treadmill. **(B)** Number of recorded running trials for each treadmill speed for all three treadmill grades. This information was used for balancing the dataset.

Measurements were conducted with 34 healthy subjects (age: 35 ± 11 years; height: 174 ± 10 cm; weight: 69 ± 12 kg; max. aerobic speed: 16.89 ± 2.81 km/h) on a motorized treadmill (T-170-FMT, Arsalis, Belgium). Ethical approval for the study was obtained from the Human Research Ethics Committee (CER-VD 2015-00006), and prior written consent was obtained from all the participants. The treadmill was customized to enable an adjustable inclination and incorporated a force plate with 3-D force recording at 1,000 Hz. The participants were equipped with IMUs (Physilog 5, GaitUp, Switzerland) attached to the shoelaces using rubber clips. Acceleration (±16 g) and angular velocity (±2,000 deg/s) were recorded at 512 Hz and were calibrated according to Ferraris et al. (1995) before each measurement session. The participants were allowed to wear their personal running shoes. Figure 1A illustrates this sensor setup.

The treadmill running protocol included four sessions with different combinations of treadmill speed and gradient, which were separated enough (≥1 week) to allow recovery in between. The first session aimed to evaluate participants’ fitness, based on ventilatory threshold and VO_2max_ assessments using an incremental speed test. As an incentive, each participant received an evaluation of his/her running performance (ventilatory thresholds and VO_2max_ level) and running technique. In sessions 2, 3, and 4, the participants went through a series of 4-min running bouts at different running speeds (8, 10, 12, and 14 km/h) and slope gradients (0%, ±5%, ±10%, +15%, and ±20%). The order of these conditions was shuffled between sessions in order to remove experimental bias. We used VO_2max_ assessments to personalize the different running conditions (speeds and slopes) within each session to avoid excessive fatigue of the participants (Morgan and Daniels, 1994; McGawley, 2017) and prevent a bias in the measurements. Among all the participants, 100% (34) completed the first session, 88% (30) completed the second session, 79% (27) completed the third session, and 71% (24) completed the fourth session. Figure 1B shows a reduction in participation with the increasing physical intensity of the conditions, which mainly corresponded to an increasing treadmill speed and grade. This led to an imbalanced dataset, with low-intensity runs being represented more.

### 2.2 Reference power estimation

**FIGURE 2:**
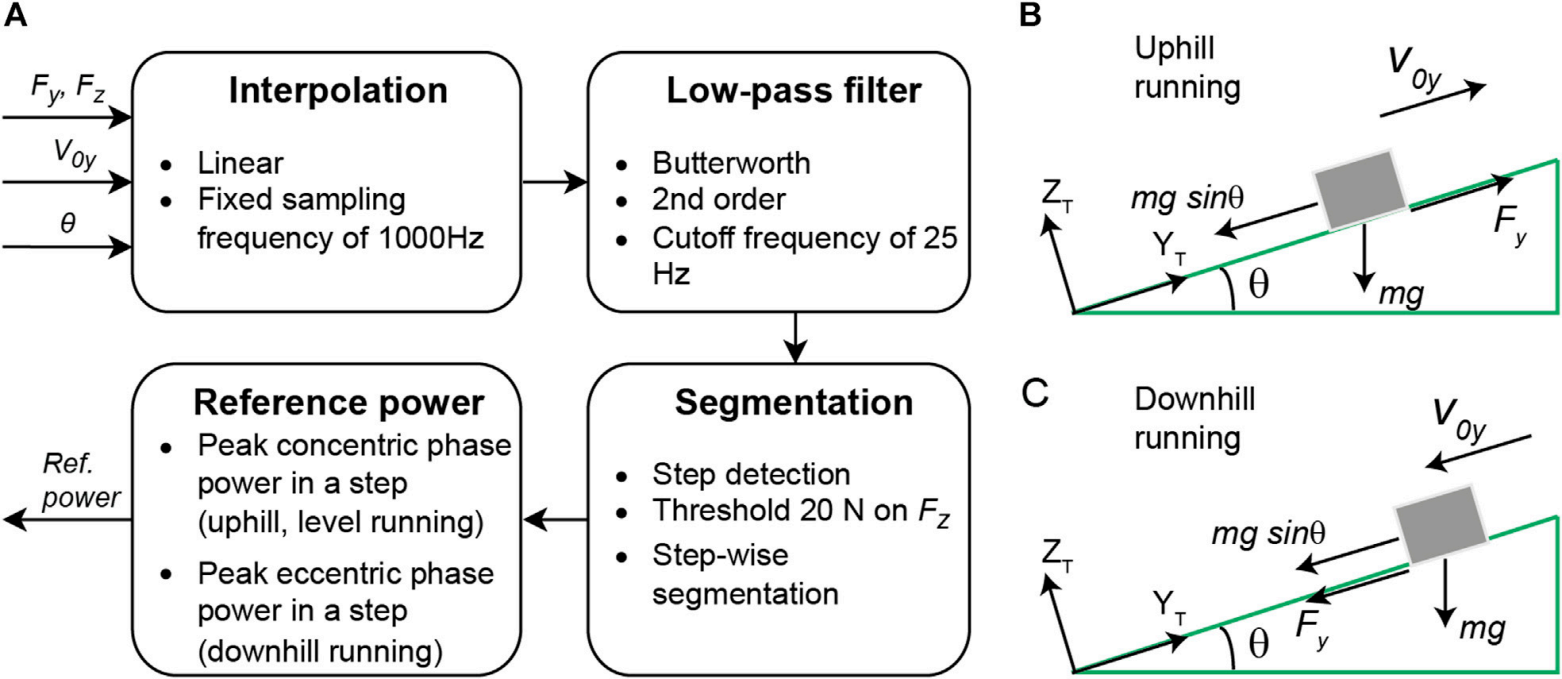
Estimation of reference horizontal power. **(A)** Processing force plate data. **(B)** Free body diagram for up-hill running on the treadmill, with the runner represented as a single rigid body (point mass). **(C)** Free body diagram for downhill running.

The procedure for estimating the reference horizontal power is shown in Figure 2A. Force plate signals along the sagittal plane, in the direction of running ( $F_{y}$ ) and perpendicular to it ( $F_{z}$ ), were checked for outliers, and linear interpolation was used to replace the removed outliers. The signals were subsequently filtered using a zero-phase low-pass Butterworth filter of order 3 and cutoff frequency of 25 Hz, based on the recommendation of using approximately 20 Hz for matched low-pass filtering of kinematic and force plate data (Mai and Willwacher, 2019). A threshold of 20 N on the $F_{z}$ signal was used to detect the stance phase (Zeni et al., 2008). First frames with $F_{z}$ higher and lower than 20 N for a length of at least 40 samples were ascertained as initial and terminal contact. To segment the gait cycles, the signal above the threshold of 300 N was used to detect the mid-stance using the *findpeaks* function in MATLAB.

Two main approaches have been considered in literature works for the estimation of horizontal power (Arampatzis et al., 2000): first is based on the GRF and the CoM motion and second is based on the estimation of the product of force–velocity or moment–angular velocity of all the individual limb segments (Cavagna et al., 1964; Cavagna and Kaneko, 1977). The latter method requires precise 3D motion tracking of each segment and is more prone to outliers in the results. Furthermore, the first method shows a better correspondence with oxygen uptake (Arampatzis et al., 2000). In this method (Rabita et al., 2015), the antero-posterior (in the direction of the run) GRF is used to estimate the antero-posterior acceleration, velocity, and horizontal power of the CoM. This method was adapted for graded running, as illustrated in Figure 2B, using the following equations:

$$a_{y}=\frac{F_{y}-mg\sin\theta }{m},$$ (1)

$$v_{y}=v_{0y}+\int_{0}^{t}a_{y}dt,$$ (2)

$$P_{y}=v_{y}\times F_{y},$$ (3)

where y is the direction of running and $F_{y}$ is the force recorded by the force plate along y , $a_{y}$ is the instantaneous acceleration of the CoM, $v_{y}$ is the instantaneous velocity of the CoM, $P_{y}$ is the “power,” $W_{y}$ is the “work,” m is the body mass, and t is the time elapsed since the beginning of the stance phase. $v_{0y}$ is the average velocity of the CoM during running, i.e., speed of the treadmill and the slope θ is assumed to be positive. For downhill running (Figure 2C), the direction of running (direction of $F_{y}$ , $a_{y},v_{y}$ , and $v_{0y}$ ) is reversed, leading to a different equation for $a_{y}$ :

$$a_{y}=\frac{F_{y}+mg\sin\theta }{m}.$$ (4)

During the implementation, all quantities are considered as scalars since the direction ( y ) is already specified. Therefore, $P_{y}$ is not power in a strict mechanical sense, as it represents only one component of a 3-D movement. A reliable estimate of average power requires not only a reliable estimate of power but also the duration of the braking and propulsion segments of the stance phase. Therefore, estimation of average power may be prone to error when determining stance phase events. We are not aware of methods that can separate these two segments with a low error in the field using foot-worn IMUs. While previous research has linked average power over the gait cycle to metabolic power (Grabowski and Kram, 2008; Taboga et al., 2021), our application mainly focuses on the feedback of running intensity and propulsive performance. Thus, we considered peak values of power as they do not require segmentation of the stance phase. To improve the robustness of the method, we estimated the peak values within one gait cycle and averaged them over multiple cycles. Therefore, in case of level and uphill running, the maximum power in the concentric phase was assumed as the reference horizontal power ( P ) value for one step (Roberts and Belliveau, 2005). For downhill running, the minimum power (negative peak) during the eccentric phase was considered the reference value ( P ) for one step. Assuming only the concentric phase power and averaging it over the stance phase (Taboga et al., 2021) is not suitable for downhill running, as it involves mainly an eccentric activation of the thigh muscles (Eston et al., 1995).

### 2.3 IMU data processing

**FIGURE 3:**
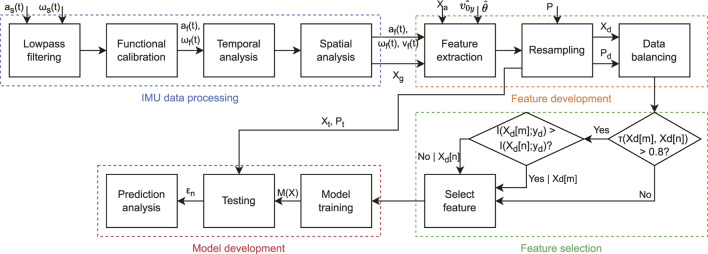
Block diagram for the proposed estimation method. The process is divided into four main parts: i) processing of the IMU signals, ii) extraction of features based on IMU signals, biomechanical and anthropomorphic parameters, treadmill speed, and slope, iii) selection of features based on reducing redundancy and maximizing the relevance, and iv) development and validation of the three models for level, uphill, and downhill running, respectively. P : reference power, $X_{d}$ : development feature set, $P_{d}$ : development set for response variable (power), $X_{t}$ : test feature set, $P_{t}$ : test set for response variable, M(x): developed model, and $\varepsilon_{n}$ : error.

The main steps for IMU data processing are shown in Figure 3. A fourth-order low-pass Butterworth filter (Fc = 50 Hz) was first applied onto the raw acceleration ( $a_{s}$ (t)) and angular velocity ( $\omega_{s}$ (t)) signals to reduce sensor noise. The filtered IMU signals were aligned with the functional frame ( $a_{f}$ (t), $\omega_{f}$ (t)) of the foot using functional calibration. The calibration process included data from a 5-s static period before the run, followed by the initial steps of the run (Falbriard et al., 2018). Following this, each signal ( $a_{f}$ (t), $\omega_{f}$ (t)) was segmented into mid-swing to mid-swing cycles, and temporal events of the gait were detected within each cycle using previously validated methods (Falbriard et al., 2018).

Due to the association between the changes in the duration of the gait phases and the running speed (Apte et al., 2021), ground contact time (*tc*), flight time (*tf*), swing time (*ts*), and stride duration (*strd*) for each step were computed using the data from the left and right foot. These temporal parameters were used as inputs for the spring-mass model to estimate vertical stiffness (*kvert*), maximum vertical force (*fzmax*), and maximum vertical displacement of the CoM (*Δz*) (Morin et al., 2005). Subsequently, we computed the orientation of the foot in the global frame (GF) X_T_-Y_T_-Z_T_ to estimate the foot strike angle (*fsa*) before initial contact and transformed the foot acceleration from the foot frame (FF) to the GF, after removing the gravitational acceleration. The resulting acceleration (in GF) was integrated using a trapezoidal rule to get a first estimate of the speed of the foot. We removed the integration drift by linearly resetting the speed at each stance phase (Falbriard et al., 2021), with the assumption of zero speed during the stance phase. Finally, we applied the inverse transformation to get the drift-corrected stride velocity of the foot segments ( $v_{f}$ (t)) in the FF that is important subsequently to develop features for the models. It is important to note that $v_{f}$ (t) is different from $v_{0y}$ , which is the treadmill speed.

### 2.4 Feature development

#### 2.4.1 Feature extraction

The overall feature development process is shown in Figure 3. The feature set consisted of four different categories of features:

1. Parameters related to gait, $X_{g}$ : The IMU-based gait spatiotemporal and stiffness parameters presented in Section 2.3 form the first feature set. The latter because vertical stiffness (*kvert)* modulates the maximum displacement of the CoM (*Δz)* in response to maximum force (*fzmax)* and has been associated with running economy, with less economical runners exhibiting a more compliant running pattern (Heise and Martin, 1998).
2. Statistical features, $X_{s}$ : Several statistical features were extracted from the $a_{f}$ (t), $\omega_{f}$ (t), and $v_{f}$ (t) signals (Table 1). Since each of signals contains three channels (x, y, and z), the statistics for each channel were calculated separately. The considered antero-posterior direction for running power was in the global frame, whereas the three directions for the IMU variables were in the sensor frame. Therefore, we assumed that the components of all three directions of the IMU would be relevant to our case and were included. In addition, the Euclidean norm was calculated for each signal, followed by the statistics for that norm. It should be noted that the statistical features ( $X_{s}$ ) were captured on the signals of a single stride, as a stride-based segmentation is more likely to capture the complete period of a gait cycle. The features $X_{s}$ aimed to encapsulate information about the signal intensity (e.g., mean, STD, and RMS), the shape of its distribution (e.g., skewness and kurtosis), and its shape in a compressed format (e.g., coefficient of the auto-regressive model). Since the temporal parameters already contain relevant periodic information, frequency domain features were not considered.
3. Anthropomorphic information, $X_{a}$ : The height, age, mass, and the leg length (measured from anterior superior iliac spine (ASIS) to the medial malleolus) of the participants were included as features. Since the mass is used in the calculation of $P_{y}$ (Eqs 1–3), it was expected to be an important feature. Leg length is related to stride length, which affects running speed. Since it is difficult to reliably estimate the stride length directly from the sensor data, we considered leg length.
4. Running conditions, $X_{c}$ : Information of the running conditions, the slope ( θ ), and the treadmill speed ( $v_{0y}$ ) were used to complete the feature set. However, to simulate real-world conditions where θ and $v_{0y}$ will be estimated from IMU and barometer signals, we added noise to known θ and $v_{0y}$ values. For $v_{0y}$ , the maximum standard deviation and bias of error for IMU-based estimation were 0.16 m/s and 0.0, respectively (Falbriard et al., 2021). So a white noise of range [-0.16, 0.16] was added to the treadmill $v_{0y}$ data before using it as a feature. Apart from this maximum noise condition (100%), two other conditions were also considered: a smaller noise (50%) of [-0.8, 0.8] and no noise, i.e., perfect estimation of $v_{0y}$ . These three conditions allowed us to explore the performance of our methods under different performances of speed estimation algorithm. We repeated the same process for θ . Assuming a 10-s window, the minimum distance estimated at the lowest treadmill speed (2.22 m/s or 8 km/h) would be (2.22–0.16) x 10 = 20.6 m. Assuming a relative height estimation error of ±1 m using a barometer (Ye et al., 2018), the error in grade was computed to be ±4.86%. So a white noise of range [−4.86, 4.86] was added to the grade data before using it as a feature. The final feature set ( $X_{c}$ ) thus consisted of the noisy speed ( $\hat{v_{0y}}$ ) and grade ( $\hat{\theta }$ ) data.

**TABLE 1:** Statistical features ( $X_{s}$ ) extracted for each stride on the continuous acceleration $a_{f}$ (t), angular velocity $\omega_{f}$ (t), and speed $v_{f}$ (t). Variables T and C correspond to the signal (a, ω, and vf) and the channel (x, y, z, or n, i.e., norm), respectively. AR, auto-regressive model.

Type	Feature	Description
Intensity	µTC	Mean value
	σTC	Standard deviation
	medTC	Median
	iqrTC	Interquartile range
	maxTC	Maximum
	rmsTC	Root mean square
Shape	kurtTC	Kurtosis
	skewTC	Skewness
Compression	arm1TC	First coefficient of the third order AR model
	arm2TC	Second coefficient of the third order AR model
	arm3TC	Third coefficient of the third order AR model

The overall feature set (X), with each feature as a vector of values, is shown as follows:

$$X=\left[X_{g},X_{s},X_{a},X_{c}\right].$$ (5)

#### 2.4.2 Resampling and dataset balancing

Because the feature set was based on segmentation of gait cycles, some inevitably misidentified gait cycles resulted in missing values. To address this problem, the data were resampled at a resolution of one value per second. Similarly, the reference power data were resampled at the same resolution and considered the response variable (P). Running conditions with a high positive grade and/or high speed had lower participation because of their high intensity, resulting in an imbalanced dataset (Figure 1B). By considering the speed as a class and dividing the grade into three conditions (level, uphill, and downhill), the classes were balanced using random over sampling (ROS) of underrepresented classes (Pes, 2020). Compared to random under sampling (RUS), ROS duplicates information rather than randomly removing samples of potentially rare conditions (e.g., high speed during uphill running). For i^th^ class with n_i_ samples, ROS was implemented as follows:

$${\overset{´}{n}}_{i}=n_{i}\times {\left(\frac{n_{\max}}{n_{i}}\right)}^{\alpha },$$ (6)

where ${\overset{´}{n}}_{i}$ is the modified sample size, $n_{\max}$ is the size of the largest class, and α is a hyperparameter. After trying values from 0.5 to 0.95 in steps of 0.05, α was set to 0.8. Finally, data from one-third of the participants (n = 11) were reserved as the test set ( $X_{t},P_{t}$ ), while the remaining data were used as the development and validation set ( $X_{d}$ , $P_{d}$ ) for the feature selection and model training phases. All data of a single participant were attributed to only one of the subsets; this removed the performance bias associated with the models trained and tested on measurements originating from the same subjects (Halilaj et al., 2018). Participants were selected randomly to form the datasets.

### 2.5 Feature selection

The feature set obtained as a result of feature extraction included a total of 171 features. To develop a simpler and more efficient model, we performed a feature selection process (Figure 3) using filter methods to remove the redundant and irrelevant features (Li et al., 2017). To identify the redundant feature pairs, we calculated the correlation between all possible feature pairs. Kendall’s τ was used to quantify the correlation between features; it is more robust than Spearman’s ρ and less sensitive to errors and discrepancies in the data (Newson, 2002). Whereas Pearson’s correlation only considers the linear relationship between variables, Kendall’s τ relies on the number of concordant and discordant pairs in the variables and does not require a specific functional relationship between variables (de Siqueira Santos et al., 2014). For feature pairs $X_{d}\left[m\right]$ and $X_{d}\left[n\right]$ with N samples, τ is quantified as

$$\tau =\frac{2}{N\left(N-1\right)}\sum_{i\;<\;j}sgn\left(X_{d}\left[m,i\right]-X_{d}\left[m,j\right]\right)\times sgn\left(X_{d}\left[n,i\right]-X_{d}\left[n,j\right]\right),$$ (7)

$$sgn\left(x\right)=\left\{\begin{array}{c} 1\forall x>0\\ 0\forall x=0\\ -1\forall x<0 \end{array}\right..$$ (8)

Feature pairs with τ < 0.8 (selected based on trials with a range from 0.5 to 0.95) were selected for model development (see Figure 3), while others were further examined for their relevance to the response variable ( $y_{d}$ ) using the mutual information ( I ) metric, which quantifies the amount of information obtained about one variable, through the availability of another variable (Kraskov et al., 2004). If $X_{d}\left[m\right]$ is considered as X and $P_{d}$ as Y , I can be expressed as

$$I\left(X;Y\right)=\sum_{y\in Y}\sum_{x\in X}p_{\left(X,Y\right)}\;\left(x,y\right)\;\log\left(\frac{p_{\left(X,Y\right)}\left(x,y\right)}{p_{X}\left(x\right)p_{Y}\left(y\right)}\right),$$ (9)

where $p_{X}$ and $p_{Y}$ are the marginal probability density function for X and Y and $p_{\left(X,Y\right)}$ is the joint probability mass function of X and Y . For feature pairs with $\tau \left(X_{d}\left[m\right],X_{d}\left[n\right]\right)$ > 0.8, feature $X_{d}\left[m\right]$ was selected if ${I(X}_{d}\left[m\right];y_{d}$ ) > ${I(X}_{d}\left[n\right];y_{d}$ ) and *vice versa* (Vergara and Estévez, 2014). The selected feature set contained 117, 125, and 120 features for level, uphill, and downhill running, with approximately 30% features being removed from each feature set. This process was not repeated for the testing set, with the selected features remaining the same.

### 2.6 Model development

#### 2.6.1 Model training

Our goal was to develop one model for each of the three running conditions. To ensure that features contributed equally to the model training and that coefficients were properly scaled, the features were rescaled using a *z*-score normalization method (Jain et al., 2005). Two approaches were pursued for model development—a linear model using elastic net (EN) regularization and a non-linear model using a neural network. Linear models enable computationally efficient implementation for near real-time analyses on conventional embedded devices. For similarly performing linear and non-linear models, EN allows us to understand the feature importance. EN linearly combines the L1 penalty of the LASSO regression method and the L2 penalty of the ridge regression method (Zou and Hastie, 2005). EN tends to maintain similar feature sparsity as the LASSO method while providing improved accuracy. Similarly, it overcomes the LASSO limitation of retaining only one of a group of linearly correlated predictors and tends to include the entire group (Zou and Hastie, 2005; Hastie et al., 2008a). The EN was implemented as shown in Eq. 10 and 11 with $P_{d,i}$ being the response at observation *i, N* being the total number of observations, $X_{d,i}$ being the feature vector with *k* features at observation *i*, λ being the positive regularization parameter corresponding to one value of lambda, β being the coefficient, and $\beta_{0}$ being the intercept.

$$\min_{\beta_{0},\beta }\left(\sum_{i=1}^{N}{\left(P_{d,i}-\beta_{0}-X_{d,i}^{T}\beta \right)}^{2}+\lambda K_{\gamma }\left(\beta \right)\right),$$ (10)

$$K_{\gamma }\left(\beta \right)=\sum_{j=1}^{k}\left(\frac{\left(1-\gamma \right)}{2}\beta_{j}^{2}+\gamma \left|\beta_{j}\right|\right),$$ (11)

where γ is the hyperparameter that sets the balance between the LASSO and ridge regression methods. Based on manual trial and error, γ was set at 0.5 for the model development. To account for interactions between biomechanical features and non-linear relationships between biomechanical parameters and “power,” a neural network (NN) was also implemented with the output layer of one neuron and a hidden layer of 10 neurons (Hastie et al., 2008b). The default MATLAB feedforward network was trained using the Levenberg–Marquardt backpropagation algorithm (Yu and Wilamowski, 2011), with a tan-sigmoid and linear transfer functions for the hidden and output layers, respectively. We trained two configurations of the NN, with differing distributions of the training dataset ( $X_{d}$ , $y_{d}$ )—NN15: 80% development, 15% validation, and 5% test; NN35: 60% development, 35% validation, and 5% test.

#### 2.6.2 Model validation and testing

The EN, NN15, and NN35 models were tested with the test set ( $X_{t},P_{t}$ ) to estimate horizontal power $\hat{P_{t}}$ . Following this, $P_{t}$ and $\hat{P_{t}}$ were smoothed by averaging over a 10-s sliding window with an overlap of 5 s. This provided a power estimate every 5 s, which we considered satisfactory for use as a running feedback tool based on our discussions with sports practitioners, while also allowing estimation of running speed $\left(\hat{v_{0y}}\right)$ and terrain grade ( $\hat{\theta }$ ) from the foot IMU and barometer signals. The estimated power $\hat{P_{t}}$ was compared to the reference power $P_{t}$ using the percentage error ( $\varepsilon_{n}$ ):

$$\varepsilon_{n}=\frac{P_{t}-\hat{P_{t}}}{P_{t}}\times 100.$$ (12)

Median and interquartile range (IQR) of $\varepsilon_{n}$ were calculated to determine the bias and precision of the power estimate. Median and IQR were also computed for each grade and treadmill speed to understand performance of the algorithm under different running conditions. The mean absolute error (MAE) was also computed using $\epsilon_{n}$ to understand the overall error. In addition, the Bland–Altman approach (Bland and Altman, 2003) was used with $P_{t}$ and $\hat{P_{t}}$ to investigate the agreement between our algorithm and the force plate-based power estimation. Finally, cumulative distribution plots of $\varepsilon_{n}$ were constructed for three noise assumptions ( $\epsilon_{100},\epsilon_{50},\epsilon_{0}$ ) on speed and slope, to provide an insight into the effects of the noise in the features on the error distribution.

## 3 Results

**FIGURE 4:**
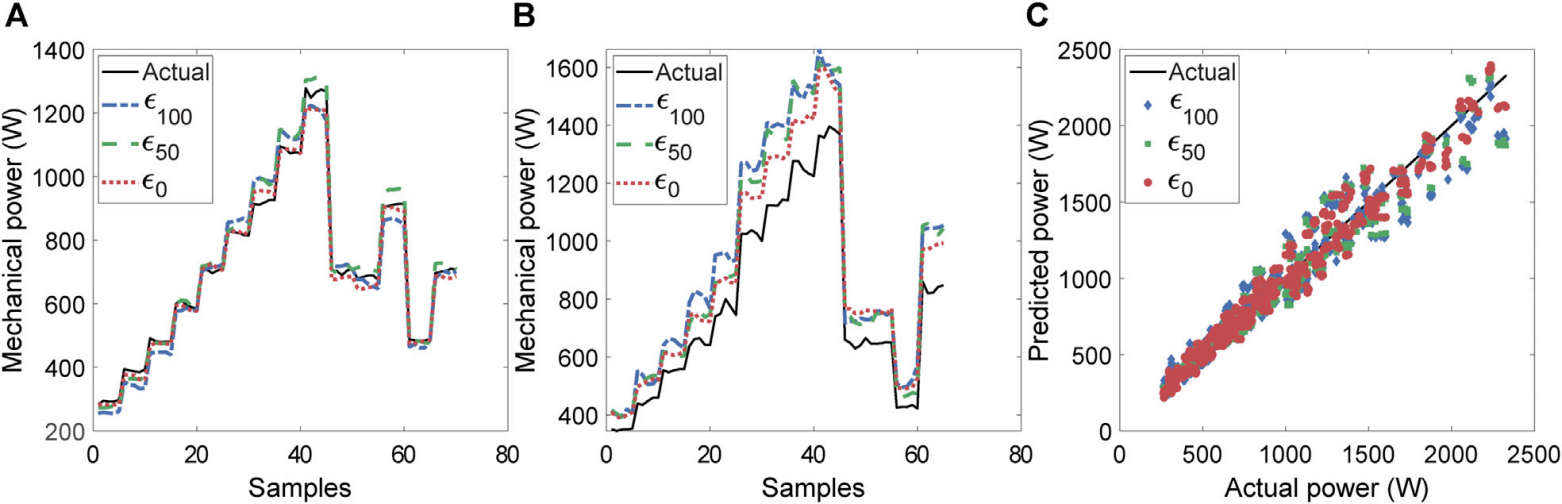
Illustration of actual and predicted horizontal power values for level running for all three noise levels on features. **(A)** Participant with the best estimation of power and **(B)** participant with the worst estimation. **(C)** Linear agreement between predicted and estimated power values.

We analyzed 34 participants who ran on a treadmill at various speeds and inclines, including a total of 210.7 min level, 74.6 min uphill, and 112.4 min downhill running, which were used for training and testing the algorithm. The reference horizontal power estimated from the force plate data followed a nearly linearly increasing relationship with treadmill speed (Supplementary Figure S1), with uphill running exhibiting a higher peak power during the concentric phase of stance than level running, at the same speed. Figures 4A, B show the best case and worst case scenarios for the prediction in level running, respectively.

The increasing power (stair pattern) corresponds to different running trials, each with higher speed than the preceding trial. The former (Figure 4A) does not exhibit a substantial difference between the prediction for the zero-noise level ( $\epsilon_{0}$ ). All three noise levels of running conditions resulted in excellent agreement (R^2^ > 0.9) between the predicted and reference values based on the linear correlation (Figure 4C). An increase in noise levels resulted in a higher deviation between predicted and reference power at high magnitudes of power. linear correlation (R^2^) between $P_{t}$ and $\hat{P_{t}}$ and the bias (median), precision (IQR), and the MAE for the error (%) are presented in Table 2. Even with the assumption of max. noise ( $\epsilon_{100}$ ), the median ± IQR for error was low (1.7% ± 12.5% for level, 3.2% ± 13.4% for uphill, and 2.0% ± 13.3% for downhill) under all three running conditions. The reduction in noise typically led to a reduction in the IQR of the error. Kendall’s test showed a high correlation (R^2^ = 0.95 for level, R^2^ = 0.91 for uphill, and R^2^ = 0.93 for downhill) between $P_{t}$ and $\hat{P_{t}}$ .

**TABLE 2:** Bias (median), precision (IQR), and mean absolute error (MAE) for the three running conditions, with different levels of noise on the features of speed and grade.

Condition	Noise	Best model	MAE (%)	Bias (%)	Precision (%)	R^2^
Level running	ϵ_100_	NN35	6.5	1.7	12.5	0.95
	ϵ_50_	NN35	6.4	3.3	10.9	0.96
	ϵ_0_	NN35	5.2	2.1	9.6	0.97
Uphill running	ϵ_100_	NN15	7.1	3.2	13.4	0.91
	ϵ_50_	NN15	6.3	−0.2	13.1	0.91
	ϵ_0_	NN35	5.4	2.4	8.9	0.95
Downhill running	ϵ_100_	EN	6.8	2.0	13.3	0.93
	ϵ_50_	NN35	6.9	2.1	11.9	0.95
	ϵ_0_	NN35	4.6	−1.9	8.4	0.97

**FIGURE 5:**
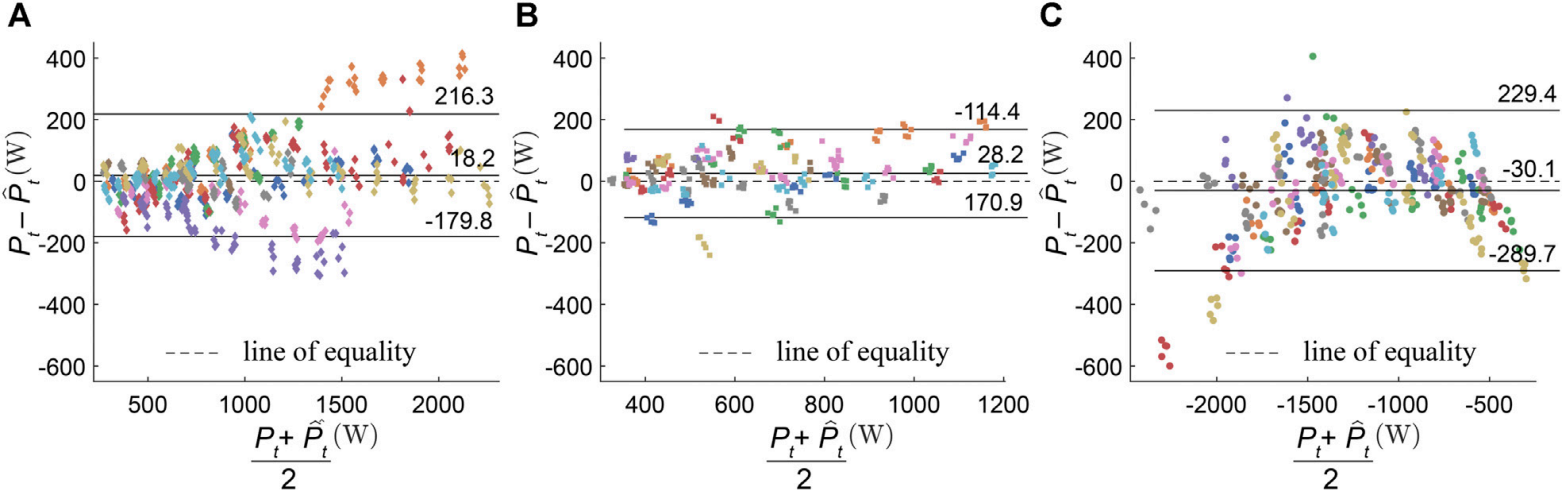
Bland–Altman analysis for horizontal power estimation with maximum noise ( $\epsilon_{100}$ ), samples from each participant in the test set are shown in different colors. $P_{t}$ and $\hat{P_{t}}$ are measured in terms of Watts (W). **(A)** Level running, **(B)** uphill running, and **(C)** downhill running.

The Bland–Altman plot for power estimation with maximum noise ( $\epsilon_{100}$ ) is presented in Figure 5, with samples from different participants represented by unique colors. It confirms low correlation between the error and estimated speed values ( τ = 0.08 for level, τ = −0.01 for uphill, and τ = 0.09 for downhill running) and an increase in error values with an increase in mean values. However, only two or three participants out of 11 show a high error at higher mean values under the three running conditions. Downhill running (Figure 5C) indicates a possible nonlinear relationship between the mean and difference of reference ( $P_{t}$ ) and estimated power ( $\hat{P_{t}}$ ) for power estimation.

The cumulative distribution of the error for all running conditions and noise levels is shown in Supplementary Figure S2. For 90% of the participants and under all conditions, the error remained below 20%, including any outliers. In contrast to level running, there was a larger influence of noisy running conditions (X_c_) on the error distribution for running on inclines. For all three running conditions, the coefficients and labels for the 15 most important features of the EN models are presented in Table 3. The most important features were usually the mass (m) and the treadmill speed ( $\hat{v_{0y}}$ ), followed by the slope ( $\hat{\theta }$ ). The linear correlation between the biomechanical features in Table 3 and the reference power for the training set is presented in Supplementary Table S1.

**TABLE 3:** Labels and coefficients for the 15 most important features of the EN models under all three conditions. Statistical features are defined according to Table 1 and are indicated in bold, with the signal direction (or norm) indicated using a subscript. Other features are defined as follows: *kvert*: vertical stiffness, *fzmax*: maximum vertical force, *Δz*: maximum vertical displacement of the CoM, *strd*: stride duration, and *fsa*: foot strike angle before initial contact. For downhill running, the negative sign indicates a positive contribution to the power estimation model since the predicted power is negative.

Level		Uphill		Downhill	
Label	Coefficient	Label	Coefficient	Label	Coefficient
$\hat{v_{0y}}$	73.8	m	48.9	$\hat{v_{0y}}$	−139
m	60.0	**iqr**a_y_	34.0	m	−97.9
*kvert*	51.5	$\hat{v_{0y}}$	32.6	$\hat{\theta }$	93.8
*fzmax*	42.8	**σ**ω_y_	−32.4	**arm1**ω_y_	67.4
**skew**v_fn_	−39.2	**µ**v_fy_	28.7	**kurt**ω_x_	−65.1
**µ**a_z_	37.4	**iqr**v_fx_	27.4	*fzmax*	−59.8
**skew**a_y_	−36.5	**kurt**a_z_	−26.2	**med**a_y_	59.1
**max**v_fn_	34.7	**µ**ay	−25.6	**σ**a_n_	−57.2
**µ**a_y_	−33.5	*strd*	19.9	*fsa*	−54.8
*Δz*	29.2	**skew**ω_x_	−19.9	**max**a_z_	52.0
**rms**a_y_	27.7	*Δz*	19.7	**rms**a_n_	−51.9
**iqr**a_y_	26.3	**max**ω_x_	−19.3	**µ**a_y_	−50.9
**arm1**v_fz_	22.4	$\hat{\theta }$	18.5	**iqr**ω_y_	50.5
**skew**v_fy_	−22.4	*lenleg*	−18.1	**arm3**ω_y_	48.9
**σ**v_fz_	21.6	**max**a_z_	−17.0	**skew**a_y_	−47.6

**FIGURE 6:**
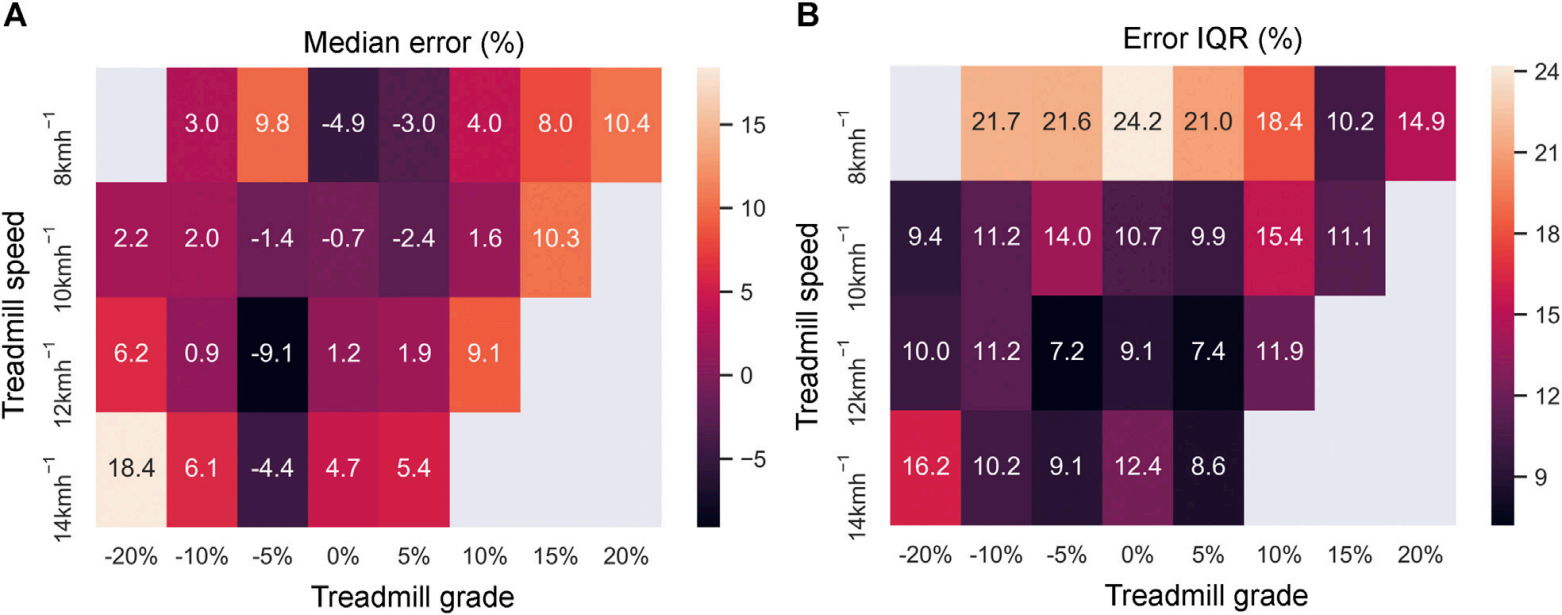
Estimation of error (%) for all running conditions. **(A)** Median error and **(B)** error IQR.

Figures 6A, B present the bias and precision for the estimation error across all treadmill speeds and slopes. For each positive slope and the −10% and −20% slopes, the bias was the largest at the highest speed reached (10.4% at 20% slope and 18.4% at −20% slope). However, the bias and the precision at the lowest treadmill speed (8 kmh^-1^) was generally high (21% ± 5.9%) at all slopes, including level running. At the 10 kmh^-1^ and 12 kmh^-1^ speed conditions, the estimation error showed a typically better precision (10.7% ± 2.0%).

## 4 Discussion

The proposed method was able to track the reference horizontal peak power estimated from the force plates over a speed range from 8 kmh^-1^ to 14 kmh^-1^ and at slopes from −20% to 20%. It achieved a MAE 6.5%–7.1%, an IQR (precision) of 12.5%–13.4%, and a R^2^ ≥ 0.91 under all running conditions (Table 2). Although obtained using only foot-worn IMUs, these error magnitudes lie within the range of RMSE values (4%–20%) obtained using a simulated full-body IMU setup (Fohrmann et al., 2019). At the same speed, uphill running exhibited a higher peak power during the concentric phase of stance than level running (Supplementary Figure S1). These results are in agreement with the findings of Arampatzis et al. (2000) who compared different methods of estimating power from kinematic and ground reaction forces (GRF) data and recommended the use of GRF data-based methods. The best models for level, uphill, and downhill running (Table 2) were the neural network with 35% validation set (NN35), the neural network with 15% validation set (NN15), and elastic net (EN) regularization, respectively.

The bias (median error) was the highest under the conditions with the highest speed and slope (Figure 6A). Running under these intense conditions is highly demanding, which limited the availability of data for model training and likely biased the models toward lower or moderate intensity running conditions. The precision (IQR for the error) at the lowest treadmill speed (8 kmh^-1^) was generally high (21% ± 5.9%). The high IQR may also be the result of the running biomechanics associated with the low speed, as 8 kmh^-1^ is within the average range of walk-to-run transition speeds (4.68–9.18 kmh^-1^) for healthy participants (Thorstensson and Roberthson, 1987). In addition to biomechanics, the higher IQR may also be the result of noise added to the speed value. Because the amount of noise was fixed, the signal-to-noise ratio (SNR) was lowest at the lowest speed (8 kmh^-1^). Considering the fact that speed is one of the most important features (Table 3) for the EN model, a low SNR can lead to a higher error. Compared to the 8 kmh^-1^ condition, the 10 kmh^-1^ and 12 kmh^-1^ speed conditions resulted in a lower IQR of error (10.7% ± 2.0%). These two conditions are within the range of average preferred running speeds in the field: 9.86 kmh^-1^ (95% CI: 9.54–10.15 kmh^-1^) for female participants and 11.7 kmh^-1^ (95% CI: 11.45–12 kmh^-1^) for male participants (Selinger et al., 2022). Furthermore, these conditions also correspond to the optimal treadmill speeds in the laboratory, which result in minimal net metabolic cost of transport (energy expenditure per unit distance travelled) for running (Selinger et al., 2022). Thus, in the context of real-life scenarios, we can expect the algorithm to perform adequately. Furthermore, it is important to note that these results are for the condition with the highest amount of noise (ϵ_100_, Table 2). With a more accurate estimation of speed and slope, we can only expect the error IQR to reduce, as is evident in the error distribution plot (Supplementary Figure S2) and Table 2.

Taboga et al. (2021) compared commercially available power meters with force plate measurements for level running only and found a good agreement (L.O.A: −154.8 to 12.6 W; M.D.: −70.8 W, assuming a reported average mass of 68.1 kg). While the upper L.O.A is lower than our findings (L.O.A: −179.8 to 216.3 W; M.D.: 18.2 W), the M.D. is higher. However, L.O.A in our case has been extended mainly due to the samples from two participants (Figure 5A). We could not find existing validation studies i^th^ graded running for comparison. As vertical force and velocity is considered for the estimation of power in case of commercial devices (Arampatzis et al., 2000; Taboga et al., 2021), hopping on the spot or increased vertical movement of the CoM during running may result in a higher power measurement. If the goal of using power as a feedback tool is to understand the intensity of the run, “power” in the direction of running (horizontal power) is a more interesting metric as it relates to the propulsion produced by the athlete (Jaskólski et al., 1996). This is despite the fact that power is a scalar quantity and “directional power” does not mechanically represent power (van der Kruk et al., 2018; Vigotsky et al., 2019). During the terminal stance phase, maximum mechanical power correlates with the push-off force generated by the concentric contraction of the thigh muscles, while maximum mechanical power absorbed during the initial contact indicates the energy absorbed by the eccentric contraction of the calf muscles (Mann and Hagy, 1980). The ability to run downhill at the same speed and gradient, but with a lower negative mechanical work, i.e., lower magnitude of “power” in the eccentric contraction phase is beneficial, as exercise-induced muscle damage during eccentric loading has a significant adverse effect on endurance performance (Marcora and Bosio, 2007). The reduction in impact forces can decrease the muscle fatigue accumulated during downhill running and possibly reduce injury risk in a trail running training program. Commercial devices only consider the power produced during the concentric phase and average this power over the entire stance phase duration, thus providing no insight into the “power” during the motion cycle. If both phases are considered together, it can lead to the averaging of positive and negative power, leading to their negation.

The EN model allows us to rank the features according to their importance (Zou and Hastie, 2005) based on the magnitude of their coefficients. We have presented the 15 most important features to explore the biomechanical contributions to the estimation of horizontal running power. However, it is important to point out that the proposed models cannot perform well by including only the 15 features presented. The list of features shows the mass (*m*) and the treadmill speed ( $\hat{v_{0y}}$ ), followed by the slope ( $\hat{\theta }$ ). This is expected due to the use of θ , $v_{0y}$ , and θ in Eqs 1–4 for the estimation of the reference power from force plate. The SNR for $\hat{\theta }$ is much lower for lower values of the gradient (e.g., ±4.86% noise for a gradient value of 5%). Compared to downhill running, uphill running has less than half the data samples at higher gradients (15 or 20%) and thus likely shows relatively much lower feature importance (Table 3) for the gradient. Biomechanical parameters such as vertical stiffness (*kvert*), maximum vertical force (*fzmax*), maximum vertical displacement of the CoM (*Δz*), stride duration (*strd*), and foot strike angle immediately before initial contact (*fsa*) were also among the important features. All these parameters showed a significant linear correlation between −0.52 and 0.82 (Supplementary Table S1) with the reference power for the training set. The magnitude of the correlation coefficient was the highest for *kvert* and *fzmax* under the level running condition and the lowest for *fsa* in downhill running. During graded running, *kvert* can exhibit large variability and there is a reasonable doubt about the validity of using the spring-mass model to estimate *kvert* under this running condition (Meyer et al., 2023). This could explain the inclusion of *kvert* as an important feature for level running but not for graded running. With an increase in speed, *Δz* decreases, *fzmax* increases, and so does the total contribution of *a_y_* and *F_y_*, leading to an increase in power (Farley and Ferris, 1998; Cavagna et al., 2005). While this implies that features are correlated, their strength of correlation (τ) was likely below the selected threshold of 0.8. *kvert*, *fzmax*, and *Δz* are directly related to the storage and return of elastic energy in the spring-mass model of running, and a decrease in kvert due to fatigue has been associated with a decrease in performance (Morin et al., 2005; 2006; Prigent et al., 2022).

### 4.1 Limitations and future work

Some of the important statistical features (Table 3) are associated with signals in the X direction, i.e., the axis perpendicular to the sagittal plane. This suggests that the 2-D model (Figures 2B, C) used to estimate reference power can be extended to account for motion in all three dimensions. In addition, this model assumes that the athlete is a point mass driven by the GRF. Although the model is mechanically driven in equilibrium (van der Kruk et al., 2018), it can be augmented to include the 3-D kinetics of the body segments. Body weight normalization of the estimated power could help compensate for variations across individuals, although weight-normalized errors would translate differently to heavier and lighter individuals. Total power, including vertical and horizontal components, has been shown to correlate with metabolic power (Arampatzis et al., 2000). If the reported total power decreases at the same running speed (with training), one can assume improved efficiency. While we only considered horizontal power in this study, our methods could be extended to estimate total power, which could be useful as feedback on running efficiency.

To enable the application of our method in practice, algorithms using accelerometer signals (Herren et al., 1999) or barometer (Moncada-Torres et al., 2014) can be devised to identify uphill, downhill, and level running. Furthermore, the ratio between the absolute power from concentric work and eccentric work could potentially be utilized as a metric of mechanical efficiency (Vernillo et al., 2017). While our model has been tested on young healthy adults running on treadmills, it can be extended further and personalized to account for different populations (Hoenig et al., 2020). In addition to foot-worn sensors, IMUs on other segments, particularly the wrist and trunk, must be examined to estimate power. Wrist location offers ease of use and has been used for running gait analysis (Kammoun et al., 2022), while the trunk provides a position close to the CoM of the body.

## 5 Conclusion

In this work, we developed a method for accurate estimation of horizontal running power with foot-worn IMUs under various simulated real-world conditions. Different inclines (−20% to 20%) and running speeds (8 kmh^-1^ to 14 kmh^-1^) were considered to test the method with the force plate data used to estimate reference power. The proposed neural network model resulted in the lowest errors (median ± interquartile range) of 1.7% ± 12.5% and 3.2% ± 13.4% for the uphill and level running, respectively, whereas the proposed elastic net model showed the lowest error of 1.8% ± 14.1% for downhill running. We accurately estimated peak concentric power for downhill running and peak eccentric power for level and uphill running, which can potentially be used to define the training load for level and trail running. Athletes susceptible to or recovering from muscle injuries can use the eccentric power peak as a threshold for designing training programs with an appropriate mechanical load. This work can provide athletes and coaches with a more comprehensive understanding through reliable in-field quantitative feedback and help further personalize training programs.

## Data Availability

The raw data supporting the conclusion of this article will be made available by the authors, without undue reservation.
