# Supplementary material for: Estimation of horizontal running power using foot-worn inertial measurement units
Source: Front Bioeng Biotechnol. 2023 Jun 22;11:1167816. doi: 10.3389/fbioe.2023.1167816 (PMC10324974; doi:10.3389/fbioe.2023.1167816)
Supplement: Supplementary file 1 [file DataSheet1.pdf]

# *Supplementary Material*

## Estimation of running power using foot-worn inertial measurement units

**Salil Apte*, Mathieu Falbriard, Frédéric Meyer, Grégoire P. Millet, Vincent Gremeaux, and Kamiar Aminian**

*** Correspondence:** Corresponding Author: salil.apte@epfl.ch

Figure S1 presents the magnitude of reference power across all treadmill speeds.

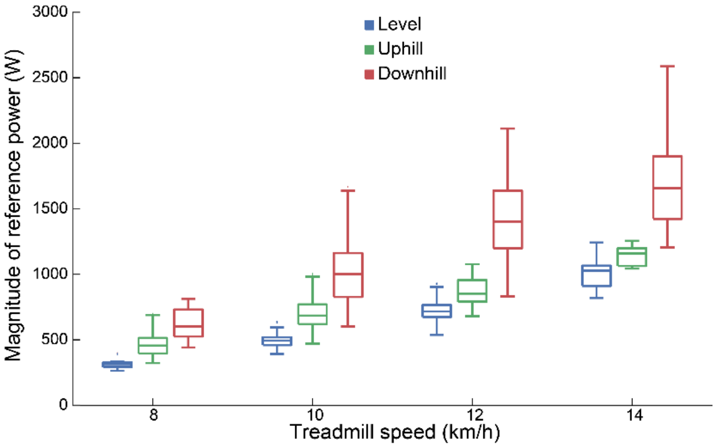


**Figure S1 Relationship between the reference power and the treadmill speed**

Figure S2 shows the cumulative distribution of the error ($\epsilon_{100}, \epsilon_{50}, \epsilon_0$) for all running conditions and noise levels. The error remains below 20% for 90% of the participants at all conditions, including any outliers. In contrast to level running, there is a larger influence of noisy running conditions ($X_c$) on the error distribution for running on inclines.

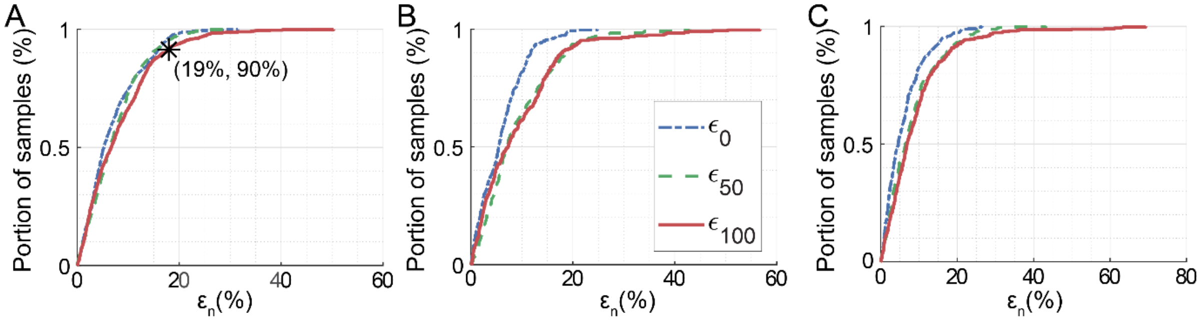


**Figure S2 Cumulative distribution of $\epsilon_{100}$, $\epsilon_{50}$, and $\epsilon_0$ (%) of the proposed method for level (A), uphill (B), and downhill (C) running. For example, in plot A, the * shows the error for level running is less than 19% for 90% of the population, when considering the 100% noise condition.**

Table S1 presents the linear (Pearson) correlation between the actual running power for the training set and the biomechanical features; these are presented within the 15 selected features (Table 3) of the manuscript.

**Table S1 Linear (Pearson) correlation for the biomechanical features. Features are defined as *kvert*: vertical stiffness, *fzmax*: maximum vertical force, *Δz*: maximum vertical displacement of the CoM, *strd*: stride duration, and *fsa*: foot strike angle before initial contact. NA indicates that this feature was not selected among the 15 features for the respective condition. * indicates significance at p<0.05**

| Selected biomechanical features (top 15) | Running condition | | |
|---|---|---|---|
| | Level | Uphill | Downhill |
| *kvert* | 0.82* | NA | NA |
| *fzmax* | 0.66* | NA | 0.33* |
| *Δz* | -0.52* | -0.42* | NA |
| *strd* | NA | -0.39* | NA |
| *lenleg* | NA | 0.43* | NA |
| *fsa* | NA | NA | 0.29* |
